# Corneal Confocal Microscopy Detects Neuropathy in Patients with Type 1 Diabetes without Retinopathy or Microalbuminuria

**DOI:** 10.1371/journal.pone.0123517

**Published:** 2015-04-08

**Authors:** Ioannis N. Petropoulos, Patrick Green, Agnes W. S. Chan, Uazman Alam, Hassan Fadavi, Andrew Marshall, Omar Asghar, Nathan Efron, Mitra Tavakoli, Rayaz A. Malik

**Affiliations:** 1 Centre for Endocrinology and Diabetes, Institute of Human Development, University of Manchester and Central Manchester National Health System Foundation Trust, Manchester Academic Health Science Centre, Manchester, United Kingdom; 2 Weill Cornell Medical College Qatar, Division of Research, Qatar Foundation, Education City, Doha, Qatar; 3 Queen Mary's University, Bart's and London National Health System Trust, London, United Kingdom; 4 Institute of Health and Biomedical Innovation and School of Optometry and Vision Science, Queensland University of Technology, Brisbane, Australia; Hirosaki University Graduate School of Medicine, JAPAN

## Abstract

**Objective:**

Corneal innervation is increasingly used as a surrogate marker of human diabetic peripheral neuropathy (DPN) however its temporal relationship with the other microvascular complications of diabetes is not fully established. In this cross-sectional, observational study we aimed to assess whether neuropathy occurred in patients with type 1 diabetes, without retinopathy or microalbuminuria.

**Materials and Methods:**

All participants underwent detailed assessment of peripheral neuropathy [neuropathy disability score (NDS), vibration perception threshold (VPT), peroneal motor nerve conduction velocity (PMNCV), sural sensory nerve conduction velocity (SSNCV) and in vivo corneal confocal microscopy (IVCCM)], retinopathy (digital fundus photography) and albuminuria status [albumin: creatinine ratio (ACR)].

**Results:**

53 patients with Type 1 diabetes with (n=37) and without retinopathy (n=16) were compared to control subjects (n=27). SSNCV, corneal nerve fibre (CNFD) and branch (CNBD) density and length (CNFL) were reduced significantly (p<0.001) in diabetic patients without retinopathy compared to control subjects. Furthermore, CNFD, CNBD and CNFL were also significantly (p<0.001) reduced in diabetic patients without microalbuminuria (n=39), compared to control subjects. Greater neuropathic severity was associated with established retinopathy and microalbuminuria.

**Conclusions:**

IVCCM detects early small fibre damage in the absence of retinopathy or microalbuminuria in patients with Type 1 diabetes.

## Introduction

Diabetes and its complications represent a growing global health burden, affecting over an estimated 366 million people worldwide [1]. The triad of retinopathy, nephropathy and neuropathy are well recognised microvascular complications, and are the leading causes of premature blindness, end-stage renal failure, foot ulceration and amputation, respectively [2]. Once established, they have a major impact on the quality of life of patients with diabetes and are associated with adverse healthcare outcomes [3].

Diabetic retinopathy (DR) is strongly associated with nephropathy [4], and is one of the earliest microvascular complications [5]. However, recent studies have shown that early neuronal abnormalities, such as altered multi-focal electroretinogram (mfERG) responses [6], retinal nerve fibre layer thinning [7] and loss of central visual field sensitivity [8] occur before the onset of overt vascular lesions in the retina, and may be of prognostic value. Krolewski et al. have previously found a strong association between cardiac autonomic neuropathy (CAN) and proliferative DR (PDR) in patients with type 1 diabetes suggesting an underlying etiologic link [9]. Indeed, the Rochester Diabetic Neuropathy Study has shown that markers of microvessel damage such as DR and proteinuria or microalbuminuria (MA) are the strongest predictors for the severity of DPN [10].

Recently, corneal nerve morphology assessed by IVCCM has been proposed as an early surrogate marker for small nerve fibre damage in DPN [11, 12]. Furthermore, corneal nerve fibre length correlates with clinical and electrophysiological measures of diabetic peripheral neuropathy [13, 14] and long term glycaemic control [15, 16]. Recent studies have shown a stepwise deterioration in corneal nerve morphology in healthy subjects and patients with pre-proliferative and PDR [17, 18]. The exact temporal relationship between neuropathy and retinopathy or nephropathy however remains unclear. The purpose of this cross-sectional, observational study was to establish whether neuropathy determined using the highly sensitive techniques of IVCCM and neurophysiology was present in patients with Type 1 diabetes without retinopathy or microalbuminuria.

## Materials and Methods

### Study subjects

80 subjects in total (53 with type 1 diabetes and 27 controls) were assessed in this study. Participants were excluded if they had a positive history of malignant, connective tissue or infectious disease, deficiency of vitamin B_12_ or folate, chronic renal failure, liver failure, active diabetic foot ulcers, family history of peripheral neuropathy. Participants were also excluded if they had active ocular disease (except for DR), systemic disease known to affect the cornea other than diabetes or chronic corneal pathologies. Inclusion criteria for patients with diabetes mellitus were a previous clinical diagnosis of type 1 diabetes, confirmed by laboratory biochemistry, and age between 18–85 years old. Controls were confirmed not to have diabetes also by laboratory testing and were of the same age range.

### Ethics Statement and data availability

This study adhered to the tenets of the Declaration of Helsinki and was approved by the North Manchester Research Ethics Committee. Study subjects with diabetes were recruited from the Manchester Diabetes Centre and control subjects were recruited from the community or were relatives of the subjects with diabetes. Informed written consent was obtained from all subjects prior to participation after explanation of the nature and possible consequences of the study. The full, anonymised dataset can be found at https://researchdata.ands.orf.au/longitudinal-assessment-neuropathy-markers-landmark/461294.

### Clinical assessment

All study participants underwent assessment of HbA_1c_ (%), lipid fractions [total cholesterol (TC) (mmol/l), high (HDLC) and low density lipoprotein cholesterol (LDLC) (mmol/l), triglycerides (mmol/l)] and albumin to creatinine ratio (ACR) (mmol/l).

### Peripheral Neuropathy assessment

The modified neuropathy disability score (NDS) was assessed [19]. VPT was tested on the hallux using a Neuroesthesiometer (Horwell, Scientific Laboratory Supplies, Wilfrod, Nottingham, UK). Electro-diagnostic studies were undertaken using a Dantec “Keypoint” system (Dantec Dynamics Ltd, Bristol, UK) and peroneal motor and sural sensory nerves [peroneal motor nerve amplitude (PMNCV), sural sensory nerve amplitude (mV) and peroneal motor nerve conduction velocity (PMNCV), sural sensory nerve conduction velocity (SSNCV) (m/s)] were assessed in the left lower limb (calf-to-ankle) by a consultant neurophysiologist.

### In-Vivo Corneal Confocal Microscopy

All study subjects were scanned with a laser IVCCM [Heidelberg Retinal Tomograph III Rostock Cornea Module (Heidelberg Engineering GmbH, Heidelberg, Germany)]. This IVCCM uses a 670 nm wavelength helium neon diode laser, which is a class I laser and therefore does not pose any ocular safety hazard. A 63x objective lens with a numerical aperture of 0.9 and a working distance, relative to the applanating cap (TomoCap^©^, Heidelberg Engineering GmbH, Heidelberg, Germany) of 0.0 to 3.0 mm was used. The size of each two-dimensional image produced was 384 μm x 384 μm which has a 15° x 15° field of view and 10 μm/pixel transverse optical resolution. This type of IVCCM uses an entirely digital image capture system and all images are stored in an external hard drive.

A drop of 0.4% benoxinate hydrochloride (Chauvin Pharmaceuticals, Chefaro, UK) was used to anaesthetise each eye and Viscotears (Carbomer 980, 0.2%, Novartis, UK) were used as the coupling agent between the cornea and the applanating cap. All subjects were asked to fixate on an outer fixation light throughout the IVCCM scan and a CCD camera was used to image the cornea and correctly position the applanating cap onto the corneal apex. The overall examination took approximately 5 minutes for both eyes of each subject and in this study two experienced optometrists performed all IVCCM scans. All images were captured using the “section” mode in the Heidelberg Explorer of the HRT III RCM. 5 to 8 images provide an acceptable level of accuracy to quantify the corneal subbasal nerve morphology [20]. We selected and analysed 6 high clarity images/subject from the central subbasal nerve plexus. Criteria for image selection were depth, focus position and contrast.

### Image Analysis

One examiner masked from cardiometabolic, peripheral neuropathy and retinopathy status quantified subbasal nerve morphology in 480 images of all study participants, using semi-automated, purpose-written, proprietary software (CCMetrics, M. A. Dabbah, Imaging Science Biomedical Engineering, University of Manchester, Manchester, UK). The specific parameters measured per frame were those we have previously established [12]: CNFD (no./mm^2^), CNBD (no./mm^2^) and CNFL (mm/mm^2^). Specific details on the definition and measurement of each parameter can be found elsewhere [21].

### Study definition for retinopathy and nephropathy

The grade of retinopathy for patients with diabetes was defined as the most recent result (1 or more examinations per annum) obtained from the UK NHS Diabetic Eye Screening Programme (the full scope of the programme can be accessed here: http://diabeticeye.screening.nhs.uk/service-specification), which is responsible for providing nationwide retinal screening for patients with diabetes mellitus (the latest DR grading criteria as set by the Royal College of Ophthalmologists can be found at:http://www.rcophth.ac.uk/news.asp?itemid=1016&itemTitle=DIABETIC+RETINOPATHY+GUIDELINES+ADDED&section=24&sectionTitle=News). Patients with “background retinopathy” or greater were classified as “with retinopathy”. To rule out retinopathy in control subjects, a standard field fundus photograph was captured using a Canon CR-2 Plus digital, non-mydriatic retinal camera (Canon Healthcare Technologies, Melville, New York, USA) and the image was graded by a study certified optometrist using the criteria proposed by the Early Treatment of Diabetic Retinopathy Study [22]. To be eligible for the study, control subjects (determined by assessment of their cardio-metabolic status and medical history) had to have a gradable ocular fundus image in at least one eye. If signs of retinopathy were found the participant was informed and excluded from the study. Albuminuria was determined using the albumin-to-creatinine ratio (ACR) and microalbuminuria (MA) in this study was defined as an ACR >2.5 mg / mmol in males and >3.5 mg / mmol in females. Similarly, control subjects with an abnormal ACR were informed and excluded from the study.

### Statistical analysis

Statistical analysis was performed using SPSS for Windows XP (Version 16.05.0, IBM, NY, USA) and graphs were generated with OriginPro for Windows XP (Version 8.5.0 SR1, OriginLab Corporation, Northampton, MA, USA). All data are expressed as mean ± SEM. Variables were tested for normality by means of univariate analysis, histograms and the Shapiro-Wilk test. One-way analysis of variance or non-parametric Kruskal Wallis was used to the test for differences between the means. Post-hoc analysis (Tukey) was performed and after correction for multiple testing (Bonferroni) a P<0.05 was considered significant.

## Results

### Clinical assessment

Detailed clinical and demographic results are presented in Table 1. Briefly, diabetic patients (duration 28.6 ± 2.2 years) compared to control subjects had similar age (49.7 ± 2.1 v 49.7 ± 2.3, P>0.05) and BMI (27.0 ± 0.6 v 27.9 ± 0.9), higher HbA1c (%) (8.3 ± 0.3 v 5.6 ± 0.1, P<0.001), a significantly lower TC (4.2 ± 0.2 v 5.2 ± 0.2, P<0.001), LDLC (2.1 ± 0.1 v 3.0 ± 0.1, p<0.001), serum triglycerides (1.2 ± 0.1 v 1.7 ± 0.2, P<0.005) and comparable HDLC (1.6 ± 0.2 v 1.5 ± 0.1, P>0.05).

**Table 1:** Demographic results for study participants stratified according to retinopathy and nephropathy status.

*Group*	Age	T1D Duration (Years)	HbA1c (%)	BMI (kg/m^2^)	ACR (mg/mmol)	TC (mmol/mol)	HDLC (mmol/mol)	LDLC (mmol/mol)	Trigs (mmol/mol)
**Controls** (n = 23)	49.7 ± 2.1	N/A	5.6 ± 0.1	27.9 ± 0.9	0.8 ± 0.1	5.2 ± 0.2	1.5 ± 0.1	3.0 ± 0.1	1.7 ± 0.2
**Diabetes No DR** (n = 17)	43.5 ± 13.6	19.2 ± 4.0	8.0 ± 0.3†	26.5 ± 1.3	0.9 ± 0.2	4.3 ± 0.3†	1.5 ± 0.1	2.2 ± 0.3†	1.1 ± 0.2†
**Diabetes DR** (n = 36)	52.3 ± 15.8‡	32.7 ± 2.4‡	8.4 ± 0.3†	27.1 ± 0.7	4.0 ± 1.4‡ †	4.1 ± 0.1†	1.6 ± 0.1	2.0 ± 0.2†	1.3 ± 0.1†
**Diabetes No MA** (n = 39)	46.9 ± 2.9	25.7 ± 2.9	8.1 ± 0.2*	27.5 ± 1.8	0.9 ± 0.1	4.1 ± 0.2*	1.6 ± 0.1	2.0 ± 0.8*	1.1 ± 0.2*
**DiabetesMA**(n = 14)	55.9 ± 4.8**	35.1 ± 4.2**	8.8 ± 0.8* **	25.3 ± 1.4	6.1 ± 2.0* **	4.3 ± 0.3*	1.6 ± 0.1	2.2 ± 0.8	1.3 ± 0.1*

Results are expressed as mean ± SEM. For patients with diabetes stratified according to retinopathy status:

^†^ significantly different from controls,

^‡^ significantly different from “diabetes without DR”.

For patients with diabetes stratified according to albuminuria status:

* significantly different from controls,

** significantly different from “diabetes without MA”.

A *P* <0.05 was considered significant.

### Neuropathy assessment

Detailed neuropathy results for patients stratified according to their retinopathy or microalbuminuria status are presented in Table 2.

**Table 2:** Neuropathy measurements in controls and patients with Type 1 diabetes stratified according to retinopathy or microalbuminuria status.

*Group*	NDS	VPT (V)	SSNA (μV)	SSNCV (m/s)	PMNA (μV)	PMNCV (m/s)	CNFD (no./mm^2^)	CNBD (no./mm^2^)	CNFL (mm/mm^2^)
**Controls** (n = 23)	0.4 ± 0.2	6.8 ± 1.1	16.4 ± 1.7	50.2 ± 0.8	5.4 ± 0.4	48.1 ± 0.6	37.1 ± 1.3	101.7 ± 7.4	27.7 ± 1.1
*Neuropathy in patients with and without diabetic retinopathy*									
**Diabetes No DR** (n = 17)	1.8 ± 0.7	8.7 ± 2.1	11.8 ± 1.5	45.9 ± 1.6 †	7.8 ± 3.1	44.8 ± 0.8	28.1 ± 2.3 †	56.1 ± 6.9 †	20.6 ± 1.5 †
**Diabetes DR** (n = 36)	3.5 ± 0.5 †	17.7 ± 2.2 †	7.3 ± 1.0 †	42.3 ± 0.9 ‡	3.3 ± 0.7 †	39.0 ± 1.2 †	22.1 ± 1.2 ‡	49.9 ± 4.8	17.4 ± 0.9
*Neuropathy in patients with and without microalbuminuria*									
**Diabetes No MA** (n = 39)	2.1 ± 0.5 *	10.9 ± 1.6	9.9 ± 1.2	44.5 ± 1.0	5.7 ± 1.7	42.7 ± 1.0	26.3 ± 1.5 *	56.7 ± 5.5 *	19.9 ± 1.7 *
**Diabetes MA**(n = 14)	5.2 ± 1.0 **	25.0 ± 4.7 **	5.5 ± 1.6 *	40.5 ± 1.7 *	1.8 ± 0.5	35.5 ± 2.4 *	17.3 ± 2.0 **	37.9 ± 7.0 **	14.3 ± 1.7 **

Results are expressed as mean ± SEM. For patients with diabetes stratified according to retinopathy status:

^†^ significantly different from controls,

^‡^ significantly different from “diabetes no DR”.

For patients with diabetes stratified according to albuminuria status:

* significantly different from controls,

** significantly different from “diabetes no MA”.

A *P* <0.05 was considered significant.

### Neuropathy vs. retinopathy

**Fig 1:**
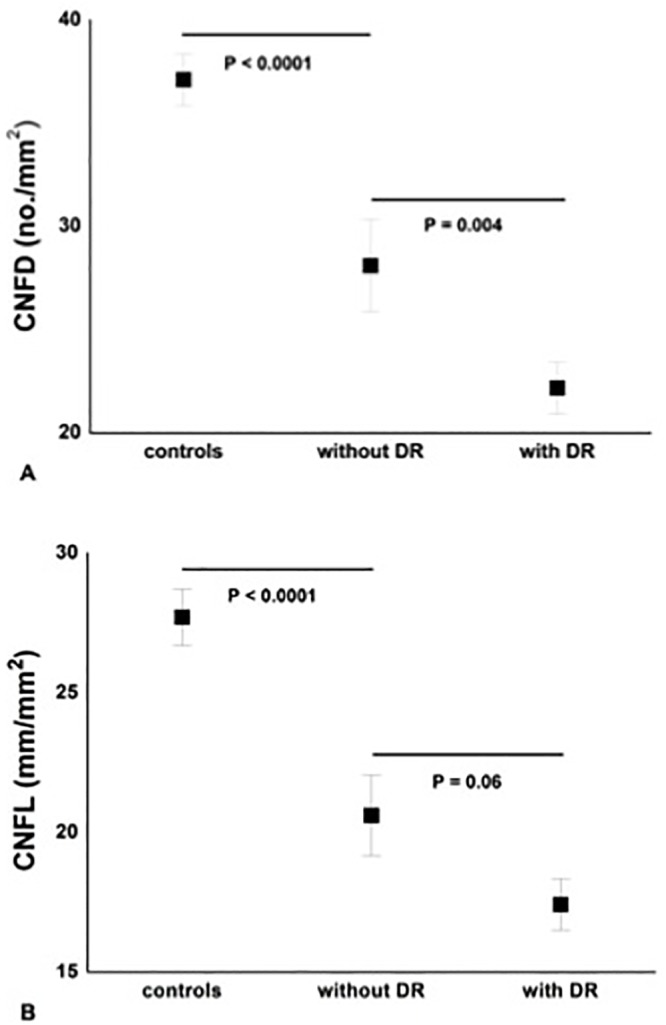
Significant and progressive loss of CNFD (A) and CNFL (B) between controls, diabetic patients ‘without DR’ (n = 17) and ‘with DR’ (n = 36). Results are expressed as mean ± SEM.

**Fig 2:**
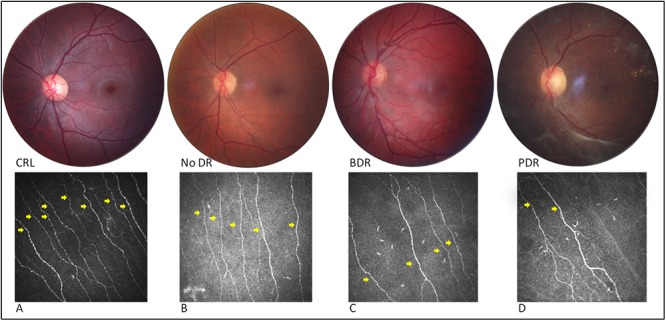
Fundus photograph of the central 30° with corresponding IVCCM image of the central subbasal nerves (yellow arrows) for control (non-mydriatic) (CTR) (A) and patients with diabetes and varying stages of DR (B, C and D). From left to right: (A) IVCCM image shows abundant corneal nerve axons for a control without retinopathy, B) significant decrease of subbasal nerves in a patient with diabetes ‘without DR’ (No DR), C) slight progressive loss of subbasal nerves in a patient with diabetes and background DR (BDR) and D) severe axonal loss on IVCCM in a patient with diabetes and pre-proliferative DR (PDR).

Patients without DR compared to control subjects showed a significantly lower CNFD (P = 0.0001), CNBD (P<0.0001), CNFL (P<0.0001) and SNCV (P<0.05). There was a further significant reduction in CNFD between patients with and without DR (P = 0.004) (Figs 1 and 2 and Table 2). There was an inverse correlation between the retinopathy grade and CNFD (r = -0.67, P<0.001), CNBD (r = -0.58, P<0.001) and CNFL (r = -0.66, P<0.001).

### Neuropathy vs. microalbuminuria

**Fig 3:**
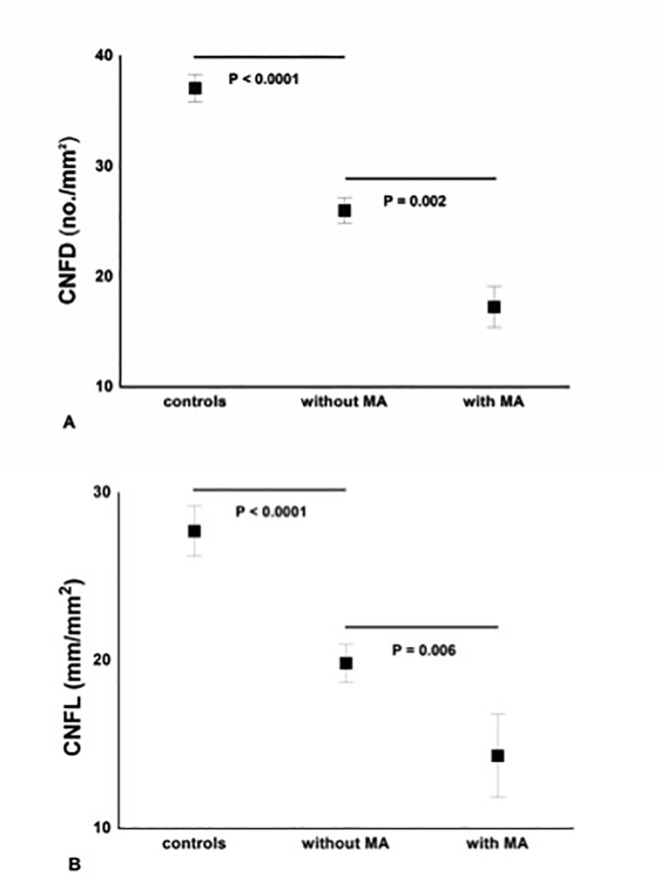
Significant and progressive loss of CNFD (A) and CNFL (B) between controls, diabetic patients ‘without MA’ (n = 39) and ‘with MA’ (n = 14). Results are expressed as mean ± SEM.

Diabetic patients without MA compared to control subjects had a significant reduction in CNFD (P<0.0001), CNBD (P = 0.001) and CNFL (P<0.0001). There was a further significant reduction for CNFD (P = 0.002), CNBD (P = 0.02), CNFL (P = 0.006) and PMNA (P<0.001) in patients with MA (Fig 3 and Table 2).

## Discussion

Diabetic microvascular complications result in considerable morbidity and both microalbuminuria and the severity of DR relate to the severity of DPN [10]. However, the temporal relationship for the development of the microvascular complications has been systematically assessed in very few studies. The United Kingdom Prospective Diabetes Study estimated the prevalence of DPN, defined as loss of vibration perception, at ~7% [23], DR at 35–39% [24] and MA at 7% at diagnosis of Type 2 diabetes [25]. In another population based study of newly diagnosed patients with Type 2 diabetes, DPN was present in 19%, retinopathy in 5% and nephropathy in 37%-48% [26]. A major limitation of these studies is the use of variable definitions for neuropathy, which are subjective and lack sensitivity, and the use of measures which detect neuropathy at an advanced stage compared to more standardized measures of retinal and renal disease, which detect these complications at an earlier stage of disease.

Over the past decade IVCCM has emerged as a novel non-invasive technique to identify early neuropathy [11–14, 27]. The loss of corneal sub-basal innervation detected using IVCCM correlates with peripheral nerve dysfunction, intra-epidermal nerve fiber [13] and retinal nerve fiber layer [7] loss. Furthermore, neuroretinal abnormalities, such as altered mfERG response waveforms, occur in the absence of retinopathy [6] and recently loss of central visual field sensitivity has been related to the severity of DPN [8]. Importantly, prospective evaluation of mfERG responses has shown that signal alterations can predict by precise location impending retinal vasculopathy and/or edema. Previously, the Retinopathy in the Chronic Renal Insufficiency Cohort Study found a strong relationship between DR and urine protein concentration but did not assess the relationship with DPN [4].

The present study shows that IVCCM detects a significant reduction in CNFD, CNBD and CNFL in diabetic patients without DR. This is an important observation as it suggests that neuropathy may precede detectable retinopathy, consistent with recent findings from other centres [17, 18], but also that worsening of retinopathy from no DR to PDR is paralleled by further corneal nerve loss and significant peripheral nerve dysfunction. Previously, a strong link has been found between CAN and PDR [9]. Of pathophysiological relevance, retinal vessels are devoid of sympathetic innervation and depend entirely on blood flow autoregulation, which has been found defective even in diabetic subjects without retinopathy [28] and is further impaired with increasing blood flow [29].

The present study also demonstrates a significant reduction in CNFD, CNFL and CNBD and significant electrophysiological evidence of neuropathy in Type 1 diabetic patients ‘without MA’, but the observed associations were less strong than with retinopathy, especially in subjects ‘with MA’. Only 14 subjects were classified as having ‘MA’ compared to 36 ‘with DR’. Moreover, these subjects with MA showed more marked alterations in electrophysiology, clinical testing and IVCCM than those with DR. This suggests that incipient nephropathy may represent a relatively late microvascular complication, which occurs after significant neuropathy and retinopathy have developed [30]. Furthermore, differences may be attributed to the use of urine protein concentration, a measure of glomerular function as opposed to fundus photography and IVCCM both measures of structural damage.

At present retinal photography and microalbuminuria are the endpoints of choice for screening for retinopathy and nephropathy, respectively, and yet the monofilament test, a quick bedside exam, is used for neuropathy screening. This study shows that significant small nerve damage detected using IVCCM is detected in the absence of retinopathy and microalbuminuria. Therefore this challenges the current paradigm for screening strategies deployed to detect the microvascular complications of diabetes. The identification of neuropathy using IVCCM may represent the earliest window of opportunity to intervene and prevent the progression of the triad of microvascular complications. A limitation of this study is the cross-sectional design and the relatively small number of subjects studied. Another limitation is that this study assessed only patients with type 1 diabetes and therefore results may not be directly applicable to all patients with diabetes. However, two recent studies [17, 31] have demonstrated similar findings in patients with type 2 diabetes. Future, prospective studies are needed to establish the exact temporal relationship between the development of neuropathy, retinopathy and nephropathy, and to define the predictive value of IVCCM.
